# Ethyl 5-methyl-7-phenyl-1,2,4-triazolo[4,3-*a*]pyrimidine-6-carboxyl­ate

**DOI:** 10.1107/S1600536814010113

**Published:** 2014-05-17

**Authors:** Omaima M. AboulWafa, Ahmed M. Farghaly, Mohamed Teleb, Khaled S. Sinoussy

**Affiliations:** a Department of Pharmaceutical Chemistry, Faculty of Pharmacy, Alexandria University, Alexandria 21521, Egypt; b National Institute of Oceanography and Fisheries, Alexandria University, Alexandria 21556, Egypt

## Abstract

In the title compound, C_15_H_14_N_4_O_2_, the triazolo­pyrimidine ring system is almost planar (r.m.s. deviation = 0.02 Å) and the phenyl ring is inclined to its mean plane by 42.45 (9)°. The carboxyl group is inclined to the triazolo­pyrimidine ring mean plane by 57.8 (3)°. In the mol­ecule, there is a short C—H⋯O contact involving the carbonyl O atom and an H atom of the adjacent methyl substituent. In the crystal, neighbouring mol­ecules are linked by C—H⋯O hydrogen bonds, forming chains propagating along [010]. There are also weak π–π inter­actions present involving the pyridine and phenyl rings of neighbouring chains [inter­centroid distance = 3.8580 (16) Å].

## Related literature   

For information on annelated pyrimidine derivatives as promising vasodilating agents, see: Jeanneau-Nicolle *et al.* (1992 ▶); Ali *et al.* (2011 ▶). For details concerning triazolo­pyrimidines having anti­hypertensive and diuretic activity, see: Ali *et al.* (2011 ▶). For details of Biginelli di­hydro­pyrimidine calcium channel blockers, see: Rovnyak *et al.* (1995 ▶); Triggle & Padmanabhan (1995 ▶); Ohno *et al.* (2002 ▶). For potential *ex vivo* calcium-channel-blocking activity, see: Farghaly *et al.* (2013 ▶).
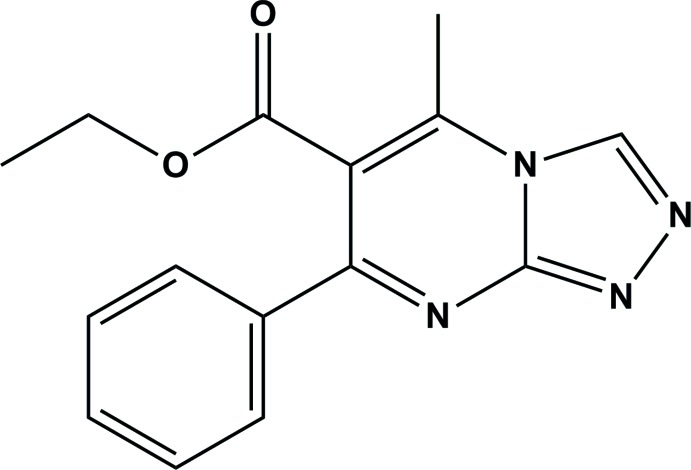


## Experimental   

### 

#### Crystal data  

C_15_H_14_N_4_O_2_
*M* *_r_* = 282.30
Monoclinic, 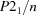

*a* = 10.322 (2) Å
*b* = 8.1678 (19) Å
*c* = 16.798 (4) Å
β = 92.111 (4)°
*V* = 1415.2 (5) Å^3^
*Z* = 4
Mo *K*α radiation
μ = 0.09 mm^−1^
*T* = 293 K
0.30 × 0.10 × 0.10 mm

#### Data collection  

Rigaku SCXmini diffractometer
Absorption correction: multi-scan (*REQAB*; Jacobson, 1998 ▶) *T*_min_ = 0.610, *T*_max_ = 0.991
12066 measured reflections
2550 independent reflections
1742 reflections with *I* > 2σ(*I*)
*R*_int_ = 0.092

#### Refinement  

*R*[*F* ^2^ > 2σ(*F* ^2^)] = 0.076
*wR*(*F* ^2^) = 0.182
*S* = 1.07
2550 reflections
193 parameters
H-atom parameters constrained
Δρ_max_ = 0.46 e Å^−3^
Δρ_min_ = −0.51 e Å^−3^

### 

Data collection: *PROCESS-AUTO* (Rigaku, 1998 ▶); cell refinement: *PROCESS-AUTO*; data reduction: *CrystalStructure* (Rigaku Americas and Rigaku, 2007 ▶); program(s) used to solve structure: *SIR88* (Burla *et al.*, 1989 ▶); program(s) used to refine structure: *SHELXL2013* (Sheldrick, 2008 ▶); molecular graphics: *PLATON* (Spek, 2009 ▶) and *CrystalStructure* (Rigaku Americas and Rigaku, 2007 ▶); software used to prepare material for publication: *SHELXL2013* and *CrystalStructure*.

## Supplementary Material

Crystal structure: contains datablock(s) global, I. DOI: 10.1107/S1600536814010113/su2724sup1.cif

Structure factors: contains datablock(s) I. DOI: 10.1107/S1600536814010113/su2724Isup2.hkl

Supporting information file. DOI: 10.1107/S1600536814010113/su2724Isup3.cml

CCDC reference: 1000732

Additional supporting information:  crystallographic information; 3D view; checkCIF report

## Figures and Tables

**Table 1:** Hydrogen-bond geometry (Å, °)

*D*—H⋯*A*	*D*—H	H⋯*A*	*D*⋯*A*	*D*—H⋯*A*
C9—H9*B*⋯O2	0.96	2.58	3.127 (4)	116
C15—H15⋯O2^i^	0.93	2.57	3.246 (3)	129

Symmetry code: (i) 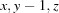.

**Fig. 1.**
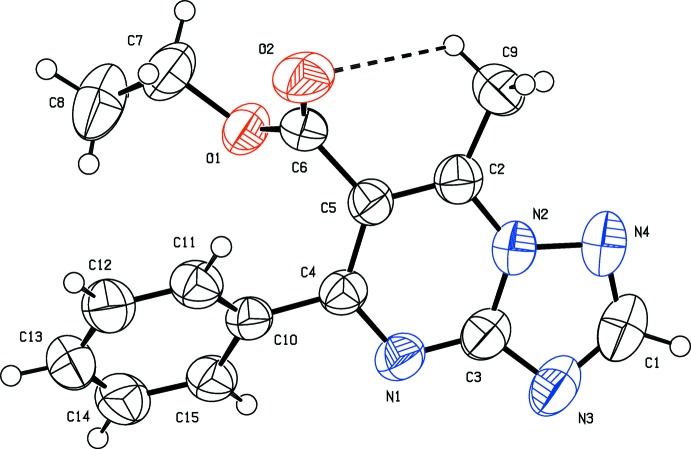
A view of the molecular structure of the title molecule, with atom labelling. Displacement ellipsoids are drawn at the 50% probability level. The intramolecular C—H···O hydrogen bond is shown as a dashed line (see Table 1 for details).

**Fig. 2.**
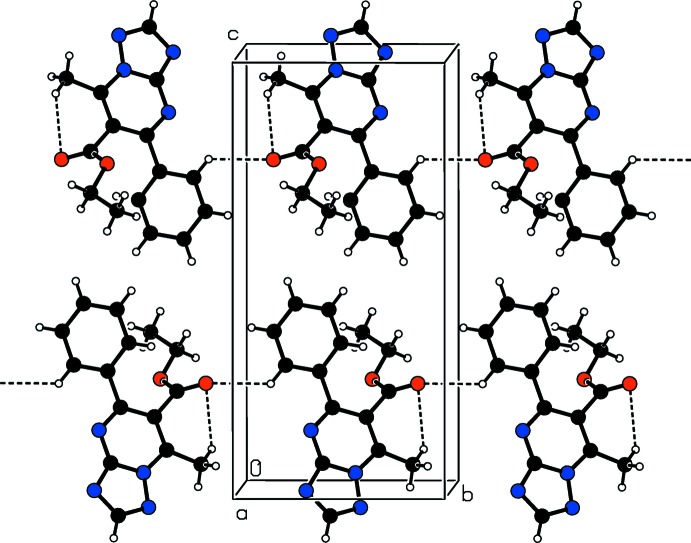
A view along the a axis of the crystal packing of the title compound. The C—H···O hydrogen bonds are shown as a dashed line (see Table 1 for details).

## supplementary crystallographic information

### 1. Introduction

Annelated pyrimidine derivatives have gained considerable inter­est as promising vasodilating agents (Jeanneau-Nicolle *et al.*, 1992; Ali *et al.*, 2011). Triazolo­pyrimidines in particular have shown anti­hypertensive as well as diuretic activities (Ali *et al.*, 2011). The title compound has a triazolo­pyrimidine nucleus and was derivatized from the well known Biginelli di­hydro­pyrimidine calcium channel blockers (Rovnyak *et al.*, 1995; Triggle & Padmanabhan, 1995; Ohno *et al.*, 2002). It has been shown to possess potential ex vivo calcium channel blocking activity (Farghaly *et al.*, 2013). In the present investigation, the crystal structure of the title compound was obtained in an effort to gain information pertaining to the role of regiocontrol in the cyclization step as well as electronic induction in the conformation of such a molecule.

### 2. Discussion

The stereochemistry of the title compound, Fig. 1, revealed that the product has retained its original stereochemistry, and that cyclization has occurred on N^1^ as predicted from HMBC (Heteronuclear Multiple Bond Correlation experiment). A close contact between the ester moiety and the phenyl group is also evident.

In the title compound, the triazolo­pyrimidine ring system is planar [r.m.s. deviation 0.02 Å] and its mean plane is inclined to the phenyl ring (C10—C15) by 42.45 (9) °. The carboxyl group (COO) is inclined to the triazolo­pyrimidine ring mean plane by 57.8 (3) °. It occupies a *cis* position relative to C5═C6 double bond of the triazolo­pyrimidine ring system thus allowing potential stabilization of such a conformation. There is a short C—H···O contact involving atom O2 and an H atom of the adjacent methyl substituent (C9), see Table 1. The ester group is again oriented away from the phenyl ring substituent for steric considerations with a torsional angle C4—C5—C6—O1 = -56.1 (3)°.

In the crystal, neighbouring molecules are linked by C—H···O hydrogen bonds forming chains propagating along [010]; Table 1 and Fig. 2. There are also weak π-π inter­actions present involving the pyridine and phenyl rings of neighbouring chains [Cg2—Cg3^i^ = 3.8580 (16) Å; Cg2 and Cg3 are the centroids of rings N1/N2/C2—C5 and C10—C15, respectively; symmetry code: (i) -x+3/2, y+1/2, -z+1/2].

### 3.1. Synthesis and crystallization

A solution of ethyl 2-hydrazino-6-methyl-4-phenyl­pyrimidine-5-carboxyl­ate (0.27 g, 1 mmole) in formic acid (5 ml) was heated under reflux for 27 h. The reaction mixture was concentrated to a small volume and diluted with ice-cold water. The precipitate was filtered, washed with water and dried. Colourless crystals of the title compound were obtained by slow evaporation of a solution in aqueous ethanol.

### 3.2. Refinement

The H atoms were included in calculated positions and treated as riding atoms: C—H = 0.93 - 0.98 Å with U_iso_(H) = 1.5U_eq_(C-methyl) and = 1.2U_eq_(C) for other H atoms.

### Crystal data

**Table:**

C_15_H_14_N_4_O_2_	*F*(000) = 592
*M**_r_* = 282.30	*D*_x_ = 1.325 Mg m^−^^3^
Monoclinic, *P*2_1_/*n*	Mo *K*α radiation, λ = 0.71075 Å
*a* = 10.322 (2) Å	Cell parameters from 10163 reflections
*b* = 8.1678 (19) Å	θ = 3.1–27.6°
*c* = 16.798 (4) Å	µ = 0.09 mm^−^^1^
β = 92.111 (4)°	*T* = 293 K
*V* = 1415.2 (5) Å^3^	Prism, colourless
*Z* = 4	0.30 × 0.10 × 0.10 mm

### Data collection

**Table:**

Rigaku SCXmini diffractometer	1742 reflections with *I* > 2σ(*I*)
ω scans	*R*_int_ = 0.092
Absorption correction: multi-scan (*REQAB*; Jacobson, 1998)	θ_max_ = 25.3°, θ_min_ = 3.2°
*T*_min_ = 0.610, *T*_max_ = 0.991	*h* = −12→12
12066 measured reflections	*k* = −9→9
2550 independent reflections	*l* = −20→20

### Refinement

**Table:**

Refinement on *F*^2^	Hydrogen site location: inferred from neighbouring sites
Least-squares matrix: full	H-atom parameters constrained
*R*[*F*^2^ > 2σ(*F*^2^)] = 0.076	*w* = 1/[σ^2^(*F*_o_^2^) + (0.1047*P*)^2^] where *P* = (*F*_o_^2^ + 2*F*_c_^2^)/3
*wR*(*F*^2^) = 0.182	(Δ/σ)_max_ < 0.001
*S* = 1.07	Δρ_max_ = 0.46 e Å^−^^3^
2550 reflections	Δρ_min_ = −0.51 e Å^−^^3^
193 parameters	Extinction correction: *SHELXL2013* (Sheldrick, 2008), Fc^*^=kFc[1+0.001xFc^2^λ^3^/sin(2θ)]^-1/4^
0 restraints	Extinction coefficient: 0.118 (11)

### Special details

**Table:**

Geometry. All e.s.d.'s (except the e.s.d. in the dihedral angle between two l.s. planes) are estimated using the full covariance matrix. The cell e.s.d.'s are taken into account individually in the estimation of e.s.d.'s in distances, angles and torsion angles; correlations between e.s.d.'s in cell parameters are only used when they are defined by crystal symmetry. An approximate (isotropic) treatment of cell e.s.d.'s is used for estimating e.s.d.'s involving l.s. planes.

### Fractional atomic coordinates and isotropic or equivalent isotropic displacement parameters (Å^2^)

**Table:**

	*x*	*y*	*z*	*U*_iso_*/*U*_eq_	
O1	0.39205 (15)	0.6185 (2)	0.24879 (9)	0.0632 (5)	
O2	0.51731 (19)	0.8413 (2)	0.24434 (12)	0.0817 (6)	
N1	0.68299 (18)	0.3481 (2)	0.14373 (12)	0.0619 (6)	
N2	0.65225 (18)	0.5557 (3)	0.04523 (11)	0.0602 (6)	
N3	0.7530 (2)	0.3301 (3)	0.00856 (15)	0.0811 (7)	
N4	0.6793 (2)	0.5809 (3)	−0.03304 (12)	0.0763 (7)	
C1	0.7390 (3)	0.4421 (4)	−0.04963 (18)	0.0844 (9)	
H1A	0.7702	0.4229	−0.1000	0.101*	
C2	0.5898 (2)	0.6607 (3)	0.09427 (14)	0.0571 (6)	
C3	0.6971 (2)	0.4037 (3)	0.06930 (15)	0.0631 (7)	
C4	0.6228 (2)	0.4455 (3)	0.19354 (13)	0.0514 (6)	
C5	0.5725 (2)	0.6021 (3)	0.16969 (13)	0.0512 (6)	
C6	0.4935 (2)	0.7029 (3)	0.22516 (14)	0.0557 (6)	
C7	0.3199 (3)	0.6916 (4)	0.31288 (18)	0.0855 (9)	
H7A	0.3792	0.7412	0.3520	0.103*	
H7B	0.2621	0.7759	0.2917	0.103*	
C8	0.2453 (3)	0.5624 (4)	0.3499 (2)	0.1047 (11)	
H8A	0.1806	0.5220	0.3123	0.157*	
H8B	0.2040	0.6054	0.3957	0.157*	
H8C	0.3023	0.4746	0.3660	0.157*	
C9	0.5465 (3)	0.8207 (3)	0.06000 (17)	0.0752 (8)	
H9A	0.6189	0.8944	0.0591	0.113*	
H9B	0.4806	0.8665	0.0921	0.113*	
H9C	0.5122	0.8044	0.0067	0.113*	
C10	0.6102 (2)	0.3845 (3)	0.27599 (13)	0.0518 (6)	
C11	0.6315 (2)	0.4852 (3)	0.34188 (15)	0.0604 (7)	
H11	0.6588	0.5925	0.3347	0.073*	
C12	0.6127 (2)	0.4282 (3)	0.41738 (16)	0.0716 (8)	
H12	0.6269	0.4969	0.4609	0.086*	
C13	0.5726 (3)	0.2680 (4)	0.42890 (17)	0.0764 (8)	
H13	0.5570	0.2304	0.4799	0.092*	
C14	0.5560 (2)	0.1660 (3)	0.36494 (18)	0.0735 (8)	
H14	0.5313	0.0579	0.3728	0.088*	
C15	0.5757 (2)	0.2217 (3)	0.28853 (15)	0.0594 (7)	
H15	0.5659	0.1505	0.2455	0.071*	

### Atomic displacement parameters (Å^2^)

**Table:**

	*U*^11^	*U*^22^	*U*^33^	*U*^12^	*U*^13^	*U*^23^
O1	0.0658 (10)	0.0599 (10)	0.0652 (11)	−0.0005 (8)	0.0222 (8)	−0.0063 (8)
O2	0.0989 (14)	0.0453 (11)	0.1024 (15)	0.0022 (9)	0.0206 (11)	−0.0087 (9)
N1	0.0677 (12)	0.0523 (12)	0.0665 (14)	0.0044 (10)	0.0151 (10)	−0.0045 (10)
N2	0.0599 (12)	0.0686 (14)	0.0527 (12)	−0.0042 (10)	0.0113 (9)	0.0021 (10)
N3	0.0879 (16)	0.0836 (17)	0.0737 (16)	−0.0003 (13)	0.0294 (12)	−0.0176 (13)
N4	0.0772 (14)	0.0967 (18)	0.0560 (14)	−0.0106 (13)	0.0176 (11)	0.0007 (13)
C1	0.088 (2)	0.100 (2)	0.0668 (19)	−0.0110 (18)	0.0246 (15)	−0.0165 (18)
C2	0.0522 (13)	0.0566 (15)	0.0629 (16)	−0.0033 (11)	0.0078 (11)	0.0020 (12)
C3	0.0631 (15)	0.0613 (16)	0.0658 (17)	−0.0007 (12)	0.0139 (12)	−0.0080 (13)
C4	0.0514 (12)	0.0438 (13)	0.0594 (15)	−0.0020 (10)	0.0069 (10)	−0.0040 (11)
C5	0.0534 (13)	0.0459 (13)	0.0549 (14)	−0.0033 (10)	0.0091 (10)	0.0008 (10)
C6	0.0640 (14)	0.0447 (14)	0.0588 (14)	0.0064 (11)	0.0079 (11)	0.0044 (11)
C7	0.092 (2)	0.082 (2)	0.085 (2)	0.0076 (16)	0.0389 (16)	−0.0107 (16)
C8	0.110 (2)	0.115 (3)	0.092 (2)	−0.026 (2)	0.0457 (19)	−0.0211 (19)
C9	0.0748 (16)	0.0750 (19)	0.0764 (19)	0.0108 (14)	0.0102 (13)	0.0226 (14)
C10	0.0540 (13)	0.0447 (13)	0.0571 (14)	0.0039 (10)	0.0076 (10)	0.0006 (11)
C11	0.0666 (15)	0.0505 (14)	0.0639 (16)	−0.0002 (11)	−0.0017 (12)	−0.0025 (12)
C12	0.0791 (17)	0.0751 (18)	0.0602 (17)	0.0074 (14)	−0.0033 (13)	−0.0020 (14)
C13	0.0807 (18)	0.084 (2)	0.0649 (17)	0.0115 (16)	0.0091 (13)	0.0192 (16)
C14	0.0725 (17)	0.0602 (17)	0.089 (2)	0.0006 (13)	0.0147 (15)	0.0154 (15)
C15	0.0653 (14)	0.0440 (13)	0.0696 (16)	0.0002 (11)	0.0109 (12)	0.0017 (12)

### Geometric parameters (Å, º)

**Table:**

O1—C6	1.327 (3)	C7—H7B	0.9700
O1—C7	1.459 (3)	C8—H8A	0.9600
O2—C6	1.198 (3)	C8—H8B	0.9600
N1—C4	1.325 (3)	C8—H8C	0.9600
N1—C3	1.343 (3)	C9—H9A	0.9600
N2—C2	1.367 (3)	C9—H9B	0.9600
N2—N4	1.370 (3)	C9—H9C	0.9600
N2—C3	1.380 (3)	C10—C11	1.390 (3)
N3—C3	1.334 (3)	C10—C15	1.395 (3)
N3—C1	1.343 (4)	C11—C12	1.372 (3)
N4—C1	1.325 (4)	C11—H11	0.9300
C1—H1A	0.9300	C12—C13	1.388 (4)
C2—C5	1.372 (3)	C12—H12	0.9300
C2—C9	1.490 (3)	C13—C14	1.365 (4)
C4—C5	1.432 (3)	C13—H13	0.9300
C4—C10	1.482 (3)	C14—C15	1.384 (3)
C5—C6	1.506 (3)	C14—H14	0.9300
C7—C8	1.459 (4)	C15—H15	0.9300
C7—H7A	0.9700		
			
C6—O1—C7	116.0 (2)	C7—C8—H8A	109.5
C4—N1—C3	117.0 (2)	C7—C8—H8B	109.5
C2—N2—N4	127.0 (2)	H8A—C8—H8B	109.5
C2—N2—C3	123.3 (2)	C7—C8—H8C	109.5
N4—N2—C3	109.8 (2)	H8A—C8—H8C	109.5
C3—N3—C1	102.2 (3)	H8B—C8—H8C	109.5
C1—N4—N2	100.7 (2)	C2—C9—H9A	109.5
N4—C1—N3	117.9 (3)	C2—C9—H9B	109.5
N4—C1—H1A	121.0	H9A—C9—H9B	109.5
N3—C1—H1A	121.0	C2—C9—H9C	109.5
N2—C2—C5	114.7 (2)	H9A—C9—H9C	109.5
N2—C2—C9	117.3 (2)	H9B—C9—H9C	109.5
C5—C2—C9	128.0 (2)	C11—C10—C15	118.5 (2)
N3—C3—N1	128.6 (3)	C11—C10—C4	121.9 (2)
N3—C3—N2	109.4 (2)	C15—C10—C4	119.6 (2)
N1—C3—N2	122.0 (2)	C12—C11—C10	120.8 (2)
N1—C4—C5	122.2 (2)	C12—C11—H11	119.6
N1—C4—C10	116.6 (2)	C10—C11—H11	119.6
C5—C4—C10	121.20 (18)	C11—C12—C13	120.1 (3)
C2—C5—C4	120.8 (2)	C11—C12—H12	119.9
C2—C5—C6	118.2 (2)	C13—C12—H12	119.9
C4—C5—C6	120.97 (19)	C14—C13—C12	119.7 (3)
O2—C6—O1	124.5 (2)	C14—C13—H13	120.2
O2—C6—C5	124.9 (2)	C12—C13—H13	120.2
O1—C6—C5	110.6 (2)	C13—C14—C15	120.7 (3)
C8—C7—O1	108.1 (2)	C13—C14—H14	119.6
C8—C7—H7A	110.1	C15—C14—H14	119.6
O1—C7—H7A	110.1	C14—C15—C10	120.0 (2)
C8—C7—H7B	110.1	C14—C15—H15	120.0
O1—C7—H7B	110.1	C10—C15—H15	120.0
H7A—C7—H7B	108.4		
			
C2—N2—N4—C1	−179.8 (2)	C10—C4—C5—C2	177.0 (2)
C3—N2—N4—C1	−0.3 (2)	N1—C4—C5—C6	174.1 (2)
N2—N4—C1—N3	0.3 (3)	C10—C4—C5—C6	−6.1 (3)
C3—N3—C1—N4	−0.2 (3)	C7—O1—C6—O2	−11.1 (3)
N4—N2—C2—C5	178.28 (19)	C7—O1—C6—C5	170.1 (2)
C3—N2—C2—C5	−1.2 (3)	C2—C5—C6—O2	−58.0 (3)
N4—N2—C2—C9	−0.4 (3)	C4—C5—C6—O2	125.1 (3)
C3—N2—C2—C9	−179.9 (2)	C2—C5—C6—O1	120.8 (2)
C1—N3—C3—N1	−179.8 (3)	C4—C5—C6—O1	−56.1 (3)
C1—N3—C3—N2	0.0 (3)	C6—O1—C7—C8	−159.8 (2)
C4—N1—C3—N3	−179.8 (2)	N1—C4—C10—C11	137.2 (2)
C4—N1—C3—N2	0.5 (3)	C5—C4—C10—C11	−42.6 (3)
C2—N2—C3—N3	179.8 (2)	N1—C4—C10—C15	−42.9 (3)
N4—N2—C3—N3	0.2 (3)	C5—C4—C10—C15	137.3 (2)
C2—N2—C3—N1	−0.4 (4)	C15—C10—C11—C12	−3.2 (3)
N4—N2—C3—N1	−180.0 (2)	C4—C10—C11—C12	176.6 (2)
C3—N1—C4—C5	1.1 (3)	C10—C11—C12—C13	0.3 (4)
C3—N1—C4—C10	−178.75 (19)	C11—C12—C13—C14	2.2 (4)
N2—C2—C5—C4	2.6 (3)	C12—C13—C14—C15	−1.7 (4)
C9—C2—C5—C4	−178.8 (2)	C13—C14—C15—C10	−1.3 (4)
N2—C2—C5—C6	−174.28 (19)	C11—C10—C15—C14	3.7 (3)
C9—C2—C5—C6	4.3 (4)	C4—C10—C15—C14	−176.1 (2)
N1—C4—C5—C2	−2.8 (3)		

### Hydrogen-bond geometry (Å, º)

**Table:**

*D*—H···*A*	*D*—H	H···*A*	*D*···*A*	*D*—H···*A*
C9—H9*B*···O2	0.96	2.58	3.127 (4)	116
C15—H15···O2^i^	0.93	2.57	3.246 (3)	129

Symmetry code: (i) *x*, *y*−1, *z*.
